# Effect of preoperative Silodosin on facilitating access sheath placement in retrograde intrarenal surgery. A randomized controlled studys

**DOI:** 10.1080/20905998.2024.2414134

**Published:** 2024-10-10

**Authors:** Ahmed Higazy, Mohamed Samir, Ahmed AbdelGhani, A.M. Tawfeek, Ahmed Radwan

**Affiliations:** a Urology Department, Faculty of Medicine, Ain Shams University, Cairo, Egypt; b Urology Department, Egyptian National Health Insurance Hospital, Cairo, Egypt

**Keywords:** Silodosin, retrograde intrarenal surgery, flexible ureterorenoscopy, ureteral access sheath, stone

## Abstract

**Introduction:**

to evaluate the effect of preoperative Silodosin on ureteric dilatation to facilitate ureteral access sheath (UAS) placement and reduction of ureteral wall injury in retrograde intrarenal surgery (RIRS).

**Methods:**

one hundred and twenty patients with renal or ureteric stones were randomly allocated into 2 equal groups. Group A represents patients who received a 7-day preoperative single dose Silodosin before RIRS while Group B represents patients who received a placebo for the same regimen. Our primary outcome was to assess the success rate of (UAS) placement. Our secondary outcomes were to evaluate the perioperative complication rate, stone-free rate, hospital stay, and cost analysis.

**Results:**

In our study, Silodosin showed a higher success rate for (UAS) insertion compared to placebo with a statistically significant difference (p-value = 0.04). Spontaneous UAS insertion in the Silodosin group was 58.3%, which was increased with active ureteric dilatation to 98.3%. Preoperative Silodosin led to less postoperative pain and analgesics requirements without impacting postoperative hospital stay or stone-free rate. There was less ureteric injury incidence in the Silodosin group compared to placebo with a statistically significant difference (p-value = 0.002).

**Conclusion:**

Preoperative Silodosin facilitates UAS insertion with a protective role against ureteric injury compared to placebo.

## Introduction

Retrograde intrarenal surgery (RIRS) is recommended for the management of renal and upper ureteric stones up to 2 cm. With greater expertise and technological advancements, RIRS can also be used successfully in the management of stones larger than 2 cm [1].

Ureteral access sheath (UAS) was developed and utilized during RIRS to facilitate instrumental access to the upper tract, allowing multiple entries for the scope, improve visibility, and lower the intrarenal pressure during the procedure, consequently minimizing perioperative complications [2,3].

UAS insertion is associated with the risk of failure to insert or may induce ureteric injury ranging from mucosal ulceration, and ureteral wall perforation up to ureteric avulsion with forceful insertion in a tight ureter [4].

To avoid failure of UAS insertion and to minimize the ureteric complications that may develop with the forceful application that may even lead to permanent ureteric stricture, some surgeons may prefer ureteric stenting before the actual procedure. However, it is not cost-effective and may be associated with pain, infection, or other comorbidities and patient dissatisfaction [5,6].

Alpha-blockers have been used in the management of ureteric stones by causing muscle relaxation of the lower ureter and a decrease in intra-ureteric resistance. Based on this concept, some studies have evaluated the impact of alpha-blocker in lowering the ureteric resistance for safe and effective access to the ureteric lumen. Alpha blocker has been shown to provide safe access to the ureter with semirigid ureteroscopies in previous studies [7,8].

Silodosin is a highly selective α1A-adrenoceptor that plays a role in reducing the lower ureteric tone, decreasing the force of contraction and frequency of peristalsis [9].

We aimed through our study to evaluate the effect of preoperative Silodosin on reducing ureteric resistance and facilitating UAS insertion in RIRS and minimizing UAS insertion-related ureteric complications and if using Silodosin may lower the procedure’s overall cost.

## Methods

After obtaining informed consent, 120 patients with renal or upper ureteric stones of 2 cm or less in the largest dimension in CT were randomly allocated in our study into 2 equal groups with a 1:1 ratio by using a sealed envelope prepared by a separate committee by the department not involved in the conductance of the study. The first group represented patients who received silodosin 8 mg single oral tablet per day for 7 days preoperatively, while patients in the other group received a placebo for the same period. Treatment allocation was concealed from patients, surgeons, and outcome assessors.

Patients with stone burden over 2 cm in the greatest dimension in CT or those with active urinary tract infections, bleeding disorders, coagulopathies, pregnancy, known ureteric stricture, abnormal anatomy, less than 18 years old or previous ureteric open surgery were excluded from the study. In addition, patients on alpha blockers or with ureteric stents during the initial evaluation were considered not eligible to be included in the study. Two surgeons were responsible for doing all the procedures.

Preoperative patient evaluation and demographic data were obtained. Patients’ compliance to medication as scheduled was ensured before surgery and those who failed to take the medication as per protocol were excluded from our study. The patient was positioned in the lithotomy position under general anesthesia, after passing the guidewire in the ureter, a trial of non-forceful UAS insertion was done initially using the UAS over the wire (Sensor tm nitinol wire with a hydrophilic straight tip, 0.035 in × 150 cm). A 13/11 Fr UAS (Navigator™ Ureteral Access Sheath (Boston Scientific Corporation, Marlborough, USA) was used in our study. In case of successful UAS insertion, a 9.5 Fr flexible ureteroscope LithoVue (manufactured by Boston Scientific, Marlborough, MA, USA) was used and passed through the UAS for laser lithotripsy. Holmium laser litho device (Quanta System, 30 W) and a 272 fiber laser were used for stone fragmentation with laser setting (energy: 0.7 J and Frequency: 20 hz)

With the failure to apply UAS from the first attempt, ureteric dilators were used to dilate the ureter up to 14 Fr, then another trial of UAS insertion was attempted. In case of, failure to dilate the ureter and UAS insertion a DJ stent was applied, and the surgery was postponed for 2 weeks.

After we finished our procedure, a diagnostic ureteroscopy was done for all cases to evaluate the ureter for any injury or complication. Documentation of any injury encountered following UAS insertion was done using a classification system for endoscopic ureteral injuries proposed by Traxer et al [5].

Our primary outcome was to evaluate the success rate of UAS placement with or without active dilatation and the impact on the ureteric wall during application. Our secondary outcomes were to evaluate the impact on the overall perioperative complication rate, stone-free rate, hospital stay, drug-related side effects, and cost analysis.

Operative time was measured in each case from the start of the diagnostic cystoscopy till urethral catheter insertion while hospital stay was measured in days. Postoperative pain was evaluated with (Mankoski pain scale). Stone-free rate was evaluated based on a CT scan 1 month postoperative where stones more than 4 mm were considered significant residual. The overall cost of each procedure included the cost of the RIRS procedure added to the cost of medication before operations. In any case needed ureteral dilatation, the cost of dilators was added. Also, in case of complications occurred, the additional cost of management of these complications was considered. Finally, in cases of failed UAS insertion, the cost of the auxiliary procedure was added in addition to the cost of the RIRS procedure. The cost was calculated initially in Egyptian pounds and was converted to U.S. dollars for standardization.

### Ethical considerations

The study was approved by the Research Ethics Committee of the Faculty of Medicine Ain Shams University, Cairo, Egypt. (FWA000017585) with ethical approval number: MS231/2023. The study was registered at Clinicaltrial.gov, with a registration number: NCT05921370.

## Statistical analysis

The statistical software for social sciences, version 23.0 (SPSS Inc., Chicago, Illinois, USA), was used to evaluate the recorded data. Quantitative data was presented in the form of mean ± standard deviation. In the evaluation of 2 means the Independent-samples t-test was used. The chi-square and Fisher’s exact test were used to compare groups with qualitative data.

## Results

**Figure 1.**
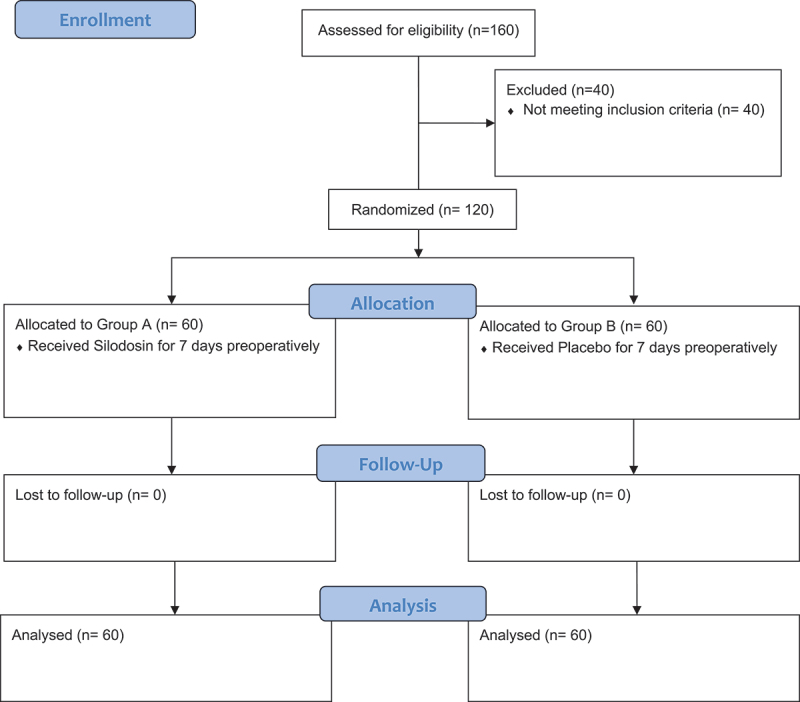
Consort flow diagram.

One hundred and twenty patients were equally distributed and evaluated for the role of Silodosin in RIRS as shown Figure 1. There was no difference in the preoperative demographic data and stone parameters as shown in Table 1.

**Table 1.** Demographic data and stone parameters.

			Group A (*n* = 60) Silodosin	Group B (*n* = 60) Placebo	P-value
Age			44.82 ± 9.53	45.47 ± 9.02	0.702
Sex	Female		18 (30.0%)	13 (21.7%)	0.297
	Male		42 (70.0%)	47 (78.3%)	0.412
BMI (Kg/m2)			29.45 ± 2	29.53 ± 3	0.860
Stone parameters	Renal		48 (80%)	45 (75%)	0.564
	Ureteric		12 (20%)	15 (25%)	0.464
	Number	Single	6 (10.0%)	4 (6.7%)	0.509
		Multiple	54 (90.0%)	56 (93.3%)	0.411
	Size (mm)		18.80 ± 4.47	17.95 ± 3.48	0.119
	HU		986 ± 313	1145 ± 246	0.075

Regarding the UAS passage in our study as shown in Table 2. In the Silodosin Group, we had a successful placement of UAS from the first attempt without dilatation in 35 patients (58.3%) compared to 4 patients in the placebo group with a statistically significant difference (P-value <0.001). Successful Placement increased with dilatation in the Silodosin group up to 98.3%. The failure rate to apply UAS in the Silodosin Group was dropped to 1.7% compared to 15% in the Placebo group with a statistically significant difference (P-value = 0.004).

**Table 2.** Evaluation of UAS passage.

		Silodosin Group (*n* = 60)	Placebo Group (*n* = 60)	P-value
Successful Placement of UAS	Spontaneous UAS Passage without dilatation	35 (58.3%)	4 (6.7%)	<0.001
	Successful Passage with dilatation	24 (40.0%)	47 (78.3%)	<0.001
	Overall UAS placement	59 (98.3%)	51 (85%)	0.004
Failed Dilatation		1 (1.7%)	9 (15%)	0.004

Perioperative parameters as shown in Table 3. There was no statistically significant difference between both groups regarding stone-free rate and hospital stay, while a statistically significant difference was seen in both operative time and cost analysis in favor of the Silodosin group.

**Table 3.** Perioperative parameters and cost analysis.

Perioperative parameters	Silodosin Group (*n* = 60)	Placebo Group (*n* = 60)	P-value
Operative time	43.77 ± 5.70	46.32 ± 5.27	0.012
Stone Residual (>4 mm)	3 out of 59 (5.1%)	3 out of 51 (5.8%)	0.231
Hospital Stay (Days)	1.22 ± 0.45	1.4 ± 0.53	0.453
Cost analysis (USD)	845.76 ± 25.13 Range: 813.4-916.2	1040.98. ± 80.13 Range: 809.4-1238.9	<0.001

The complication rate is reported in Table 4. Silodosin showed a reduction in the overall complication rate. Patients in the Silodosin group showed less postoperative pain and less requirement for postoperative analgesia. The incidence of ureteric injury was less in the Silodosin group with a statistically significant difference (P-value = 0.002). Grade 1 ureteric injury was seen in 4 patients in the Silodosin group compared to 14 patients in the Placebo group. Grade 2 complication was seen in 4 patients in the Placebo group, and none was reported in the Silodosin group. We did not encounter grade 3 or 4 ureteric injuries. No patient in our study showed Clavein Dindo classification grade 3 or 4. We did not report any significant drug-related side effects in our trial.

**Table 4.** Comparison between silodosin group and placebo group according to postoperative complications.

(Clavein Dindo Classification)			Silodosin Group (*n* = 60)	Placebo Group (*n* = 60)	P-value
Grade I	Postoperative pain (Mankoski pain scale)	0-5	45 (75%)	37 (61.7%)	0.021
		(Require extra analgesic) > 5	15 (25%)	23 (38.3%)	
Hematuria			2 (3.3%)	10 (16.7%)	0.034
Ureteral injury		Grade 1 injury	4 (6.7%)	14 (25%)	0.002
		Grade 2 injury	0	4 (6.7%)	
Grade II	UTI Requiring antibiotics		4 (6.7%)	9 (15.0%)	0.142

## Discussion

Surgeons nowadays adopt RIRS for the surgical treatment of renal and upper ureteric stones up to 2 cm and with more technological advances and the availability of cheap single-use flexible ureteroscopies, the upper limit for RIRS in the management of stone burden pushed further [10].

The value of UAS is to provide easy access to the upper urinary tract. In addition, it improves irrigation, allows clearer vision, decreases intrarenal pressure, and lowers the incidence of renal injury even though studies have stated that no significant difference in the perioperative complications with UAS [4,11–14].

UAS placement may not be smooth in all cases and sometimes it is associated with a significant risk of ureteric trauma and injury that may terminate the procedure, increase the likelihood of postoperative infection, or may be associated or a ureteric stricture in the long term. Some studies reported that a tight ureter may lead to failure of ureteroscopy in 10% of cases and UAS insertion may fail in up to 22% of cases [11,15].

Alpha blocker action on alpha-1 receptors in the distal ureter may lead to reduced intraluminal pressure and relaxation of the ureteric wall muscles of the distal ureter [16,17]. This may enhance instrumental placement into the ureter. Based on this concept, many studies evaluated the effect of perioperative alpha-blockers in facilitating access to the ureteric lumen [6,7].

In our study, we evaluated the effect of 7 days of preoperative Silodosin compared to a Placebo in RIRS. Regarding the spontaneous passage of UAS with the use of ureteric dilators, it was significantly higher in the silodosin group and the success rate increased with dilatation from 58.3% to 98.3% in the same group compared to the increase in success rate with dilatation from 6.7% to 85% in the placebo group with p (p-value = 0.004). This resulted in a decreased failure rate of UAS placement to 1.7% in the silodosin group compared to 15% in the placebo group with a statistically significant difference (p-value = 0.004).

Koo et al., in their randomized controlled trial involving 135 patients evaluating alpha-blockers for seven days preoperatively. UAS placement and the force needed for UAS insertion using alpha-blocker were significantly lower than those who did not receive preoperative alpha-blockers at the level of the ureterovesical junction with a (p-value = 0.008) and at the level of the proximal ureter (p-value = 0.036). The application force needed in the alpha-blocker group was comparable to that in pre-stented patients, thus confirming the rule of alpha-blockers in lower ureteric resistance [7].

On contrary to Erturhan et al., who evaluated Tamsulosin for 2 weeks preoperatively to RIRS. Although, UAS insertion was higher in the Tamsulosin group it failed to reach a statistically significant difference [18].

Regarding operative time in our study, it was significantly higher in the placebo group (46.32 min ± 5.27) compared to the silodosin group (43.77 min ± 5.70) (*p* = 0.012). Although it was significantly higher, it was clinically insignificant. Kopru et al. declared similar results regarding the operative time that silodosin can shorten stages of RIRS. On the other hand, a study by Shaher et al. reported that preoperative Silodosin shortens the procedural time compared to the control group with a statistically significant difference [19,20].

Our study showed that there was no statistically significant difference found between the two groups regarding both stone residual rate and hospital stay. Kim et al. reported similar results regarding the mean hospital stay and stone-free rate with no statistically significant difference between the two groups with *p* values (p = 0.972), (p = 0.874) respectively [6].

Our study evaluated the postoperative complications according to (the Clavein Dindo Classification). Postoperative pain was significantly lower in the silodosin group with less requirement for postoperative analgesia compared to placebo (*p* = 0.022). In a study by Kim et al., Silodosin reduced postoperative pain. They explained that less pain with the alpha-blocker is due to less ureteric muscle tone and less shear force with UAS insertion, as the ureteric mucosal friction with UAS stimulates nociceptors [6,21].

Ureteral mucosal injury was evaluated with a diagnostic ureteroscopy following the procedure. Grade 1 injury (ureteral mucosal erosion) was seen in 4 patients (6.7%) in the Silodosin group compared to 14 patients (25%) in the placebo group, while grade 2 injury (ureteral mucosal and smooth muscle injury) was seen in 4 patients (6.7%) in the placebo group, while it was not encountered in the Silodosin group.

Silodosin was shown to have a protective effect against UAS-induced ureteral injuries with a statistically significant difference (*p* = 0.011), and (*p* = 0.042) respectively. No cases were reported in either group for grade 3 (ureteral adventitial perforation) or grade 4 (total ureteral avulsion) according to the endoscopic classification of ureteral wall injuries by Traxer et al [5].

In a study by Kim et al. on 87 patients, Silodosin showed a significant reduction in the incidence of postoperative ureteral injury involving the ureteral smooth muscle following UAS insertion in comparison to Placebo (9.3% vs 27.3%; *p* = 0.031). However, there was no statistically significant difference regarding the overall complications [6].

Koo et al. reported in their randomized controlled trial including 135 patients that the incidence of mucosal injury (grade 2 or higher) was lower in the alpha-blocker group compared to the control group (*p* = 0.038) [7].

A systematic review and meta-analysis by Victor et al., reported that preoperative α1-blockers were associated with less incidence of ureteral wall injuries and fewer overall complications using the UAS [16].

Our study revealed that overall Cost (USD) was significantly lower among the silodosin group was 845.76 ± 25.13 compared to the control group was 1040.98. ± 80.13 with a p-value (*p* < 0.001). This could be explained by the extra need for ureteric dilators to be used in the Placebo group, and the need for passive dilatation with ureteric stent in case of failed dilation and the re-scheduling of the operation. In addition, the price of extra medication needed in the form of analgesics or antibiotics for treatment of post-operative complications.

Although cost-effectiveness was not directly assessed as in our study in RIRS between Silodosin and placebo. It was mentioned that pre-stenting was associated with lower cost-effectiveness and was associated with more peri-operative complications either irritative symptoms or urinary tract infections in previous studies [6,22,23].

The strength points of our study are that we conducted a Randomized blinded study to evaluate the role of Silodosin in RIRS and its impact on UAS insertion and we believe that we are the first to address the point of cost-effectiveness. Silodosin was used effectively to facilitate UAS insertion and decrease peri-operative complications with less UAS-induced traumatic injury to the ureter. Moreover, Silodosin was shown to be cost-effective in reducing the procedural cost in comparison to placebo.

The limitation of our study was that it was a single-centered study. The strength used by surgeons to apply the UAS is variable and can not be standardized. The follow-up period is short to detect long-term complications like ureteric stricture. However, it is unlikely with no high-grade injury to the ureter. Postoperative pain is very subjective and reported data is very variable which we tried to overcome by using a standardized scale. Flurosocopy time and radiational exposure was not assessed in our study, it less time needed to insert UAS and less need for ureteric dilatation, the radiational exposure will be less. However, it was not measured in our study and we do recommend that this point to be considered in future studies. Finally, cost-effectiveness varies from one country to another and is difficult to generalize.

## Conclusion

Preoperative Silodosin facilitates UAS insertion with less trauma to the ureteral wall in comparison to placebo.

## Data Availability

The datasets used and/or analyzed during the current study are available from the corresponding author upon reasonable request.

## Abbreviations list

RIRS retrograde intrarenal surgery
UASureteral access
